# Single-Centre Experience with the Balloon-Expandable Myval Transcatheter Aortic Valve System with the First 200 Patients: 30-Day and 1-Year Follow-Up

**DOI:** 10.3390/jcm14072323

**Published:** 2025-03-28

**Authors:** Bálint Kittka, Balázs Magyari, Ilona Goják, Gábor Kasza, Kristóf Schönfeld, László Botond Szapáry, Mihály Simon, Rudolf Kiss, Andrea Bertalan, Edit Várady, István Szokodi, Iván Gábor Horváth

**Affiliations:** 1 Heart Institute, Medical School, University of Pécs, 13. Ifjuság Str., H-7624 Pécs, Hungary; kittka.balint@pte.hu (B.K.); schonfeld.kristof@pte.hu (K.S.); szapary.laszlo.botond@pte.hu (L.B.S.); simon.mihaly@pte.hu (M.S.); kiss.rudolf@pte.hu (R.K.); bertalan.andrea@pte.hu (A.B.); szokodi.istvan@pte.hu (I.S.); ivan.g.horvath@aok.pte.hu (I.G.H.); 2 Szentágothai Research Center, University of Pécs, H-7624 Pécs, Hungary; 3 Department of Vascular Surgery, Medical School, University of Pécs, H-7624 Pécs, Hungary; kasza.gabor@pte.hu; 4 Department of Medical Imaging, Medical School, University of Pécs, H-7624 Pécs, Hungary; varady.edit@pte.hu

**Keywords:** TAVR, bicuspid aortic valve, balloon-expandable transcatheter heart valve, paravalvular leak, annular rupture, permanent pacemaker implantation

## Abstract

**Aims**: The aim of this paper is to report 30-day and 1-year outcome data regarding the first 200 patients who underwent the TAVR procedure using the Myval THV system at our single centre. **Methods**: From November 2019 to October 2022, 200 consecutive patients underwent TAVR procedure. Outcomes were analysed according to the VARC-2 definitions, and device performance was assessed via transthoracic echocardiography. Data collection was approved by the local Ethical Committee. **Results**: The mean age of the cohort was 75.3 ± 6.9 years, and 122 (61%) participants were male. The mean EuroSCORE II and STS was 5.4 ± 5.4 and 5.8 ± 3.8, respectively. The proportion of patients with a bicuspid aortic valve was 18%. The transfemoral access approach was the most common (surgical vs. percutaneous: 1% vs. 98%), and in two patients, surgical subclavian access was used. VARC-2 outcomes were as follows: 99% device success, 2% STROKE, 5% and 4.5% major and minor vascular complications, respectively, and a 29.5% rate of new permanent pacemaker implantation. At discharge, the incidence of aortic regurgitation grade II or above was 5.5% without relevant PVL (grade II or above 0.5%). In-hospital mortality was only 1%. At 1 year, the all-cause mortality rate was 8.5% (cardiac origin in three cases), and two patients had valve-related dysfunction requiring surgical aortic replacement. **Conclusions**: Our results showed excellent 30-day and 1-year outcomes regarding patient survival, technical success, and valve-related adverse events using the Myval transcatheter heart valve system. The limitations of our study comprise a single-centre design with retrospective data collection.

## 1. Introduction

Based on current guidelines, transcatheter aortic valve replacement (TAVR) therapy serves as the standard of care for patients at high and intermediate surgical risk and is an acceptable alternative to surgical aortic valve replacement (SAVR) in low-risk patients [1,2,3,4,5]. The selection between self-expandable (SEV) and balloon-expandable (BEV) transcatheter heart valve (THV) devices is still controversial. Interventionalists should consider the higher radial force of BEV devices, which results in a better PVL rate but a higher potential mechanical complication rate than SEVs, which have a better safety profile and valve performance. The novel Myval THV technology, combining the known technical advantages of BEV and SEV systems without their negative aspects, have been verified in smaller studies [6,7], and experience with this device is growing. The recently published LANDMARK trial established the non-inferiority of the Myval THV regarding 30-day safety and efficacy compared to contemporary THVs (Edwards Sapien, Medtronic Evolut) [8].

In this study, we report our early post-procedural, 30-day, and 1-year Valve Academic Research Consortium-2 (VARC-2) outcomes using the Myval THV system.

## 2. Methods

### 2.1. Study Design

This work comprised a single-centre study and the study design is similar to that in our previous report [9]. Data were recorded prospectively as the standard of care in the centralised electronic medical data collection system (e-MedSolution system) and collected retrospectively. Data collection was approved by the local Ethical Committee (9435-PTE 2022).

### 2.2. Patient Population

During the study period from November 2019 to October 2022, 200 patients were implanted with the Myval THV. As previously reported, this THV device was used exclusively in patients with a horizontal aorta or bicuspid aortic valve and when the potential benefits of an intermediate THV size could be detected based on CT scan measurements [9]. This device selection method was constant during the examined period.

Besides high-gradient severe aortic stenosis (AS) as the most common diagnosis, in patients with low-flow low-gradient aortic stenosis (LFLG-AS) and paradoxical low-flow low-gradient AS (PLFLG-AS), severity and indication were based on dobutamine stress echocardiography and/or native aortic valve CT calcium score. Patients referred for the TAVR procedure had New York Heart Association (NYHA) class II or above and were unsuitable or had a high risk for surgical aortic valve replacement (SAVR) based on the decision of the Heart Team. The logistic EuroSCORE II and STS scores were used to calculate the surgical risk. Data regarding the baseline clinical and echocardiographic characteristics of the study population are detailed in Table 1 and Table 2.

The main exclusion criteria were acute myocardial infarction within 14 days, severe left ventricular dysfunction (ejection fraction ≤ 20%), ongoing infection (including SARS-CoV-2 infection), hemodynamic instability, contraindications for antiplatelet and/or anticoagulant therapy, and a life expectancy of less than 12 months.

### 2.3. Device Description and Procedure

The technical parameters of the Myval THV (Meril Life Sciences Pvt. Ltd., Vapi, India) are well described in the MyVal-1 study. Briefly, the system comprises a bovine pericardium leaflet on a nickel–cobalt frame with anticalcification treatment, a high radial force due to the unique cell design, and internal and external sealing tissue minimising PVL. Beyond the standard sizes (20 mm, 23 mm, 26 mm, and 29 mm), intermediate (21.5 mm, 24.5 mm, and 27.5 mm) and extra-large valve sizes (30.5 mm and 32 mm) are available and compatible with the expandable 14 Fr Python sheath. Relevant data on valve sizing are shown in Table 3. Predilatation of the native aortic valve was the standard of choice, as previously reported [9].

The TAVR procedures were performed in a dedicated hybrid operating room under conscious sedation, and general anaesthesia was used in a minority of cases (two femoral and two subclavian cases). Transfemoral access was the most common, with trans-subclavian access used in two patients. When the transfemoral approach was not feasible based on the CT scan, intravascular lithotripsy therapy (IVL system, Shockwave Medical, Santa Clara, CA, USA) was applied to prepare the iliofemoral route in nine patients. During the procedure, activated clotting time (ACT) guided heparin treatment was the standard of care, followed by antiplatelet therapy (aspirin 100 mg/day and/or clopidogrel 75 mg/day). If anticoagulant therapy was needed, clopidogrel (75 mg/day) with direct oral anticoagulant (DOAC) therapy was the standard choice in case of previous percutaneous coronary intervention.

### 2.4. Study Endpoints and Follow-Up

Safety and efficacy parameters were collected before discharge and at the 1-month and 12-month follow-ups. As the primary endpoint, safety was evaluated based on periprocedural outcomes; as a secondary endpoint, the 30-day and 1-year combined safety endpoints were defined by VARC-2. Transthoracic echocardiography was used for the evaluation of the short- and long-term hemodynamic performance of this THV device. The functional status of the patients was classified based on the NYHA class. All relevant endpoints were defined according to the VARC-2 definitions [10]. The severity of perioperative aortic regurgitation was evaluated by intraoperative echocardiography, angiography, and the measurement of the aortic regurgitation index (ARI), described previously [11].

### 2.5. Statistical Analysis

GraphPad Prism (version 9.0, GraphPad Software Inc., San Diego, CA, USA) and SPSS Statistics (version 28.0, IBM, Armonk, NY, USA) were used for statistical analysis. Continuous variables are presented as mean ± standard deviation. Baseline characteristics and echocardiographic measurements were compared using a two-sample Student’s *t*-test. Binary variables were reported as percentages and compared by two-sample proportions *z*-test.

## 3. Results

### 3.1. Baseline Patient Characteristics

From November 2019 until October 2022, including the learning curve with the device, 200 consecutive patients were included in the study. The diagnosis of symptomatic AS was set using the latest European Society of Cardiology (ESC) guideline for the management of valvular heart disease. Bicuspid aortic valve anatomy appeared in 36 patients, horizontal aorta was detected in 82 patients, and previous surgical aortic or mitral bioprosthesis implantation occurred in one and two patients, respectively. The mean age was 75.3 ± 6.9 years, and 61% of participants were male. The mean logistic EuroSCORE II was 5.4 ± 5.4, and the mean STS score was 5.8 ± 3.8. The baseline clinical and echocardiographic parameters of the study population are detailed in Table 1 and Table 2.

### 3.2. Procedural Outcomes

Transfemoral access was the most common approach (99%), with two trans-subclavian implantation cases (1%). Conscious sedation was used predominantly (98%), while general anaesthesia was less frequent (2%). Overall, in 91 cases (45.7%), standard valve sizes (23, 26, and 29) were implanted, and in 108 cases (54.3%), intermediate/extra-large sizes (21.5, 24.5, 27.5, 30.5, and 32) were chosen. In patients with bicuspid aortic valves, intermediate or extra-large THVs were utilised significantly more often than standard sizes (75% vs. 25%, *p* < 0.001). Conversely, in the tricuspid aortic valve (TAV) group, the distribution of intermediate/extra-large sizes and standard sizes was nearly equal (49.7% vs. 50.3%, *p* = 0.931). The mean aortic gradient decreased significantly (53.8 ± 18.4 mmHg vs. 5.6 ± 5.3 mmHg, *p* < 0.0001).

Device success was achieved in all but two patients (99%). There was a single device failure in our learning curve with this device, wherein the unsuccessfully implanted THV device had to be removed via vascular surgery. The complications of this surgery led to a fatal outcome for the patient, as previously reported. THV malapposition occurred in one case, where after the THV was implanted, the device jumped into the left ventricle. As cardiogenic shock progressed, emergency cardiac surgery was undertaken to remove the THV device. Unfortunately, the patient died of the complications associated with the surgical intervention. Due to these two fatalities, the in-hospital mortality was 1%. Severe adverse events (including myocardial infarction, coronary obstruction, annular rupture, cardiac tamponade, the need for a second THV, or postprocedural aortic regurgitation (AR) grade III or IV) were absent. Acute kidney injury stage 2 or 3 occurred in a minority of cases (3.5%).

Although we were unable to use a cerebral embolic protection device due to funding issues, ischaemic stroke was observed only in three patients, and transient ischemic attack (TIA) occurred in only one patient. However, these neurological complications had no effect on the in-hospital mortality. At the 30-day follow-up point, one patient had died due to postprocedural stroke.

Vascular complications occurred in 19 cases; based on the VARC-2 criteria, major vascular complications (attributed to the volume of transfusions used) occurred in 12 patients and minor vascular complications were detected in seven patients. The patency of the access site could be maintained using interventional or surgical procedures in every case. Details are given in Supplementary Table S1.

Before the TAVR procedure, 17 patients already had a permanent pacemaker. New permanent pacemaker implantation (PPI) was mandatory in 54 cases, resulting in a 29.5% new PPI rate in the cohort. However, no significant differences were observed between patients who required a new PPI and those who did not in terms of age, EuroSCORE II, STS score, the calcium score of the aortic valve, and the percentage of THV sizing. On the other hand, a significantly higher EuroSCORE was observed in patients without the need for PPI. The implantation depth was measured by analysing the THV distance below the native aortic annulus after the THV implantation separately for all coronary cusps. Regarding this, excluding patients with pacemaker (PM) implantation prior to the TAVR, the average implantation depth was 5.3 ± 2.3 mm on the left coronary side, 5.8 ± 2.1 mm on the right coronary side, 6.1 ± 2.2 mm at the non-coronary side, and, on average, 5.7 ± 2.1 mm. There were no significant differences in any of the implantation depths between patients with and without a PPI. The incidence of calcification in the left ventricular outflow tract (LVOT) was significantly higher among patients who received a permanent PM (46.3% vs. 33.3%, *p* = 0.049). The existence of bicuspid aortic valve anatomy had no impact on the new PPI rate. No significant differences could be detected regarding the amount of oversizing in the comparison of patients with or without PPI (Non-PM vs. PM, 7.1 ± 4.7% vs. 7.0 ± 3.8%, *p* = 0.9). Moreover, the type of THV (standard size or intermediate/extra-large size) had no significant effect on the new PPI rate. All the relevant data regarding the procedural and postprocedural results are shown in Table 4, Table 5 and Table 6.

### 3.3. Varc-2 Outcomes at the 30-Day and 1-Year Follow-Ups

At 30 days, all-cause mortality was 2%. After patient discharge, two additional deaths occurred: one patient due to COVID-19 pneumonia and one patient due to postprocedurally evolved stroke. Overall, the 30-day cardiac mortality was 0.5%. During the 30-day follow-up period, no new strokes occurred. Life-threatening bleeding occurred in six patients, all associated with the index procedure, and only in one patient after discharge from the hospital. There was no need for intermittent regular renal replacement therapy, and coronary artery obstruction was absent. Major vascular complications did not occur after patient discharge. Due to the new onset of conduction disturbances, new PM implantation was mandatory in two patients, resulting in a 30.6% PPI rate at 30 days of follow-up. However, there was no valve-related dysfunction requiring repeated procedures; in the case of one patient, hospitalization due to the worsening of heart failure was necessary. During this period, no patient experienced functional class III, valve thrombosis, or endocarditis.

Between the 30-day and 1-year period, 13 additional deaths occurred: Five patients died due to noncardiac infections leading to multiorgan failure, two patients due to endocarditis leading to death (one patient underwent SAVR), three patients due to malignant cancer, one patient due to stroke, and two patients due to COVID-19 pneumonia acquired out of hospital. The rates of all-cause mortality and cardiac mortality at 1 year were 8.5% and 1.5%, respectively. In the follow-up period, six patients had ischaemic stroke, resulting in a 5% 1-year all-stroke rate. After the 30-day follow-up, no additional life-threatening bleeding, new onset of renal failure, coronary obstruction, or major vascular complications occurred. In this period, new pacemaker implantation was indicated in two cases: in one patient due to cardiac resynchronisation therapy and in another due to complete third-degree AV block. Valve-related dysfunction resulted in the need for surgical explantation of the TAVR in two patients. Hospitalization due to the progression of heart failure after 30 days was observed in two patients; in one patient, cardiac resynchronisation therapy pacemaker was necessary, and in another patient, the reason for admission was endocarditis of the previously implanted TAVI valve. In that patient, surgical extraction of the TAVI valve and performing SAVR was mandatory. Two patients had functional class III. However, valve thrombosis was absent, and the overall rate of prosthetic valve endocarditis at 1 year was 1.5% (three patients). In addition, one more patient had endocarditis of the native mitral valve after a urological operation, which was treated successfully.

**Figure 1:**
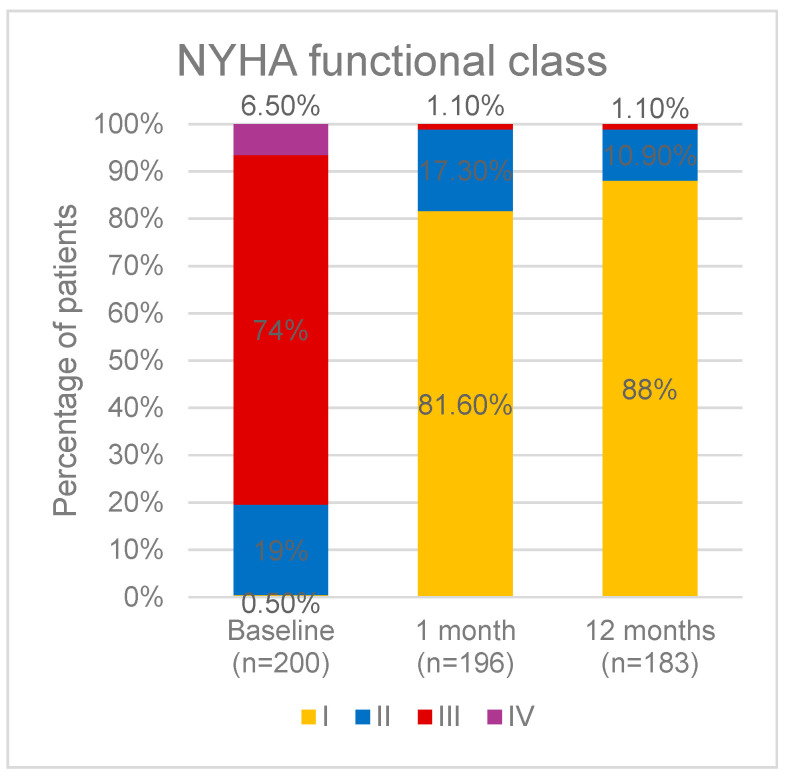
Patient distribution according to NYHA functional class regarding the different time periods of the study population. NYHA: New York Heart Association.

The patients’ functional capacity, as measured by the NYHA classification, showed significant improvement at the 30-day follow-up and continued to be maintained throughout the examined period. Only a minority of the patients were in class III, and no patient was in class IV. Details are shown in Figure 1. No patient was lost to follow-up during the examined period.

Data regarding the 30-day and 1-year outcomes are summarized in Table 7.

### 3.4. Echocardiographic Outcomes

**Figure 2:**
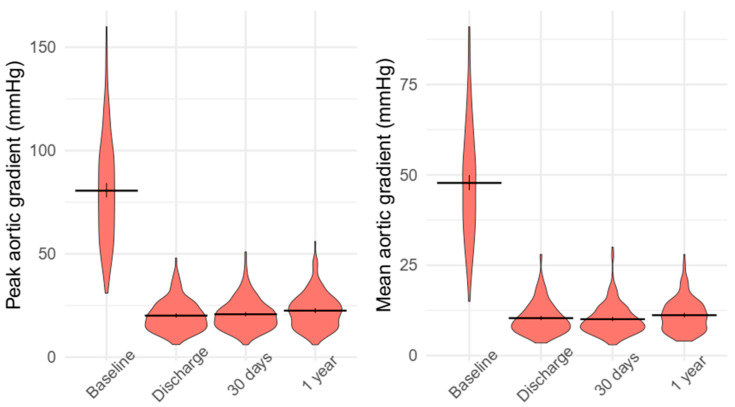
Violin plots comparing the effect of transcatheter aortic valve replacement on peak aortic valve gradient and mean aortic valve gradient measured by echocardiography in patients who survived up to 1 year.

As shown in Figure 2, there was a significant improvement in the peak aortic valve gradient after the TAVR procedure during the 1-year follow-up (*p* < 0.0001). A significant reduction in values was observed from baseline to discharge (80.6 ± 25.7 vs. 20.2 ± 7.4 mmHg, *p* < 0.0001). However, no significant change was detected from discharge to the 30-day follow-up (20.2 ± 7.4 vs. 20.7 ± 7.6 mmHg, *p* = 0.197). Between the 30-day follow-up and the 1-year follow-up, a statistically significant but minor increase was noted regarding this parameter (20.7 ± 7.6 vs. 22.5 ± 8.6 mmHg, *p* < 0.0001; Figure 2 and Table 8). A similar phenomenon could be seen concerning the mean aortic valve gradient. There was a substantial decrease between baseline and discharge values (47.8 ± 15.6 vs. 10.4 ± 4.3 mmHg, *p* < 0.0001) without relevant adjustment regarding the comparison of the discharge and 30-day follow-up values (10.4 ± 4.3 vs. 10 ± 4.2 mmHg, *p* = 0.162). As seen in terms of the peak aortic valve gradient, a statistically significant but minor increase occurred regarding aortic mean valve gradient when this parameter was compared between the 30-day and 1-year follow-ups (10 ± 4.2 vs. 11.2 ± 4.5 mmHg, *p* < 0.0001; Figure 2 and Table 8). The slight, unfavourable shift in both peak and mean aortic valve gradients was accompanied by a significant improvement in global ejection fraction over the follow-up period, rising from 55.9 ± 13.5% at discharge to 60.7 ± 10.5% at the 1-year follow-up (*p* < 0.001; Table 8).

There was a significant reduction in the percentage of patients with mitral regurgitation (MR) grade 3 or 4 after the procedure (39 [19.5%] vs. 15 [7.5%], *p* < 0.0001), which was stable during the follow-up: discharge versus 30-day follow-up (15 [7.6%] vs. 14 [7.1%], *p* = 0.317) and 30-day versus 1-year follow-up (14 [7.1%] vs. 15 [8.2%], *p* = 0.317). Similarly, the rate of patients with aortic regurgitation grade 2 or above decreased significantly (73 [36.5%] vs. 11 [5.1%], *p* < 0.0001) with further improvement regarding the comparison of discharge versus 30-day follow-up (11 [5.5%] vs. 5 [2.5%], *p* = 0.014) without any change afterwards (30-day vs. 1-year follow-up: 5 [2.5%] vs. 5 [2.7%], *p* = 1.0). It should be highlighted that during the examined period, aortic regurgitation grade 3 or 4 was absent, with an irrelevant number of patients with a moderate or above paravalvular leakage grade. Detailed data are in Table 8.

As our experiences with this THV device regarding the comparison of patients with tricuspid and bicuspid aortic valves are well described in our previous work [12], this sub-analysis was out of the scope of this report.

## 4. Discussion

Considering that compared to the LANDMARK trial, our all-comer patient cohort had a higher surgical risk profile (2.6% vs. 5.8%), a higher proportion of patients with bicuspid valves (7% vs. 18%) with a similar rate of small aortic annulus (≤430 mm^2^, 32% vs. 26%), a high device implantation rate (99%) with low in-hospital mortality rate (only 1%), and an acceptable vascular complications rate. Major mechanical complications (including cardiac tamponade, annular rupture, and left ventricle chamber perforation), valve malpositioning, and/or the need for second THV implantation were not observed. Considering this, our results are comparable with those of other studies, including the milestone LANDMARK trial [3,6,8,13,14,15], except for our high PPI rate; however, the impact of this factor is not obvious.

Remarkable haemodynamic and echocardiographic improvements were verified in our patient cohort, and this performance was stable during the examined period. Regarding the aortic mean and peak valve gradient, a statistically significant but modest increase was observed when these parameters were compared at the 30-day follow-up and 1-year follow-up. The fact that these unfavourable changes were attended by a significantly increased global ejection fraction can confirm this theory. Additionally, based on the follow-up outpatient visits, there was no need to perform multi-slice computed tomography (MSCT) examination due to the suspicion of hypoattenuated leaflet thickening or starting oral anticoagulant therapy, which can also support this hypothesis. These favourable results included a relevant portion of patients with bicuspid aortic valves.

Besides the steerable delivery system, which may be responsible for the low stroke rate, the wide sizing scale is another important advantage of the Myval THV device. This technical feature enables a more accurate patient-tailored THV choice and is very likely responsible for the lack of mechanical complications, aortic regurgitation grade 2 or above, and PVL grade moderate or above. The proportion of patients with an intermediate-size THV was more prevalent compared to those with the standard size (54.3% vs. 45.7%), but this difference was unambiguous in patients with bicuspid aortic valves (75% vs. 25%).

Considering that predilatation was the standard of choice, a cerebral protection device was not used, and postdilatation was performed at a similar rate as in the LANDMARK trial (14% vs. 10%); the postprocedural all-stroke rate (2%) is highly acceptable. Furthermore, no additional stroke occurred during the 30-day follow-up, and six new strokes evolved up to the 1-year follow-up, resulting in a 5% annual all-stroke rate.

On one hand, a modest decrease in the new PPI rate in Cohort B was detected compared to Cohort A, In the total cohort, the new PPI rate (54 cases, 29.5%) seems to be high. On the other hand, similar PPI rates have been reported in the literature [16,17,18]. Based on a report by Gonska et al., the optimal implantation depth appeared to be 4–6 mm; lower or higher implantation resulted in a higher PPI rate. Moreover, the authors found no significant differences between patients with and those without new PM implantation regarding implantation depth [19]. These findings are in concordance with our results. However, while experience with this novel, balloon-expanding THV system is growing, evidence regarding the implantation depth’s impact on the PPI rate is lacking. This study has three main findings regarding the new PPI rate. First, compared to our previous work, where only a trend could be observed between LVOT tract calcification and the new PPI rate, in this extended patient cohort, we could verify the significant effect of the existence of calcium in the LVOT tract on the new PPI rate. Second, like in our previous study, the bicuspid aortic valve anatomy did not predispose patients to new PPI using this THV system [9]. Third, except for LVOT calcification, no significant differences could be detected between patients with and those without PPI regarding the baseline characteristics, implantation technique, and used THV size (standard or intermediate/extra-large). Taking these factors into consideration, the hazard for conduction disturbances was higher in our patients receiving PPI, and, therefore, this complication seems to be more patient-related than device-related.

### What Have We Learned from the First 100 Patients?

The results of the present study align with our previous findings [9]; however, additional observations were noted when comparing the first and second cohorts of 100 patients, specifically regarding postdilatation, sizing methods, and the PPI rate.

However, predilatation was performed unexceptionally, and the rate of postdilatation in the total cohort (14%) was similar to that in the LANDMARK trial [8]; in the second 100 patients (Cohort B), this manoeuvre was significantly less frequent (25% vs. 3%, *p* < 0.001) compared to in the first 100 patients (Cohort A). On one hand, in cases where the THV implantation resulted in a suboptimal expansion, postdilatation is mandatory to reach the optimal pressure gradient and minimise the grade of PVL. On the other hand, this intervention may have a negative long-term effect [20,21]. The notably reduced rate of post-dilatation observed in Cohort B may be attributed to the enhanced experience regarding THV sizing. The lack of significant difference regarding postimplant valve gradients between cohorts and the favourable ARI index (tendency for higher postprocedural ARI in Cohort B, 27.9 ± 9.7 vs. 28.6 ± 9.1, *p* = 0.619) in addition to the lack of patients with PVL grade moderate or above in Cohort B may support this theory.

The sizing method was standard during the study period (nominal or undersized, with non-standard THV sizes), while the percentage of oversizing was lower in Cohort B than in Cohort A, although without reaching significance (6.6 ± 4.6% vs. 7.4 ± 4.0%, *p* = 0.181). This lower oversizing had no negative effect on the hemodynamic performance of the THV; moreover, a higher ARI value could be detected in Cohort B without reaching statistical significance.

However, the PPI rate remained high, and we could not find any procedural parameters that might influence this unfavourable result based on the first 100 patients [9]. In this extended patient cohort, we could verify the significant effect of the existence of calcium in the LVOT tract on the new PPI rate. Moreover, except for LVOT calcification, no significant differences could be detected between patients with and those without PPI regarding the baseline characteristics, implantation technique (no significant difference between cohorts regarding average implantation depth, Cohort A vs. Cohort B: 5.9 ± 2.1 vs. 5.9 ± 1.7, *p* = 0.947) and used THV size (standard or intermediate/extra-large size).

All data presented in this report were analysed in the context of the comparison between Cohorts A and B. These data are presented in Supplementary Tables S2–S8.

## 5. Conclusions

Based on this report, the Myval THV system demonstrated outstanding safety and efficacy with comparable results to those from studies using other THV systems and the first high-volume, randomised LANDMARK trial using this THV device. However, the new PPI rate decreased with the second 100 patients but remained high. This complication seems to be more patient-related than device-related due to the remarkably high proportion of LVOT calcification in patients with PPI, and the lack of difference regarding implantation technique between patients receiving PMs and those not. It should be emphasised that our low stroke rate was achieved without using a cerebral protection device, verifying the advantage of the steerable delivery system. The single-centre study design could limit our results. Our patient enrolment strategy may also have some selection bias; on the other hand, the high rate of bicuspid anatomy and small aortic annulus may have created a patient cohort where successful TAVR procedures can be more complex. Due to the lack of data regarding TAVR prosthesis durability with this THV system, an extended follow-up analysis is mandatory.

## Figures and Tables

**Table 1:** Baseline characteristics of the total study population. NYHA: New York Heart Association, MI: myocardial infarction, PCI: percutaneous coronary intervention, CABG: coronary artery bypass grafting, PM: permanent pacemaker, STS: Society of Thoracic Surgeons, GFR: Glomerular Filtration Rate, MVR: mitral valve replacement, AVR: aortic valve replacement.

	Overall (n = 200)	Cohort A (n = 1–100)	Cohort B (n = 101–200)	*p* Value
Age (yers)	75.3 ± 6.9	74.7 ± 7.2	75.9 ± 6.5	0.192
Male/Female	122/78	63/37	59/41	0.562
Body mass index (kg/m^2^)	29 ± 5.2	29.4 ± 4.8	28.6 ± 5.6	0.242
Body surface area (m^2^)	1.93 ± 0.2	1.94 ± 0.2	1.91 ± 0.2	0.223
Hypertension	195 (97.5%)	95 (95%)	100 (100%)	0.007
Diabetes mellitus	79 (39.5%)	40 (40%)	39 (39%)	0.885
Hyperlipidemia	182 (91%)	84 (84%)	98 (98%)	0.001
NYHA class I	2 (1%)	2 (2%)	0 (0%)	0.316
NYHA class II	39 (19.5%)	34 (34%)	5 (5%)	<0.001
NYHA class III	147 (73.5%)	60 (60%)	87 (87%)	<0.001
NYHA class IV	12 (6%)	4 (4%)	8 (8%)	0.39
Ischaemic Heart Disease	85 (42.5%)	47 (47%)	38 (38%)	0.198
Prior MI	50 (25%)	24 (24%)	26 (26%)	0.744
Prior PCI	70 (35%)	39 (39%)	31 (31%)	0.298
Prior CABG	36 (18%)	22 (22%)	14 (14%)	0.101
Peripheral artery disease	24 (12%)	10 (10%)	14 (14%)	0.384
Cerebrovascular disease	29 (14.5%)	8 (8%)	21 (21%)	0.009
Pulmonary disease	28 (14%)	15 (15%)	13 (13%)	0.836
Previous aortic balloon valvuloplasty	9 (4.5%)	5 (5%)	4 (4%)	0.733
Permanent PM	17 (8.5%)	9 (9%)	8 (8%)	0.8
Atrial fibrillation	41 (20.5%)	18 (18%)	23 (23%)	0.381
Logistic EuroSCORE (%)	15.2 ± 15	15.7 ± 15.5	15.1 ± 14.5	0.921
Euroscore II	5.4 ± 5.4	4.8 ± 4.9	6.0 ± 5.9	0.115
STS score (%)	5.8 ± 3.8	5.6 ± 3.9	5.9 ± 3.8	0.582
Aortic valve Calcium score	3308 ± 1726	3395 ± 1832	3211 ± 1628	0.437
Serum creatinine (umol/L)	102.3 ± 48.2	102.7 ± 58.8	101.8 ± 35.0	0.857
Estimated GFR (mL/min)	67.6 ± 25.9	69.6 ± 26.6	65 ± 25.5	0.224
Estimated GFR < 60 mL/min	89 (44.5%)	40 (40%)	49 (49%)	0.2
Bicuspid aortic valve	36 (18%)	17 (17%)	19 (19%)	0.713
Small annulus (≤430 mm^2^)	52 (26%)	24 (24%)	28 (28%)	0.519
Prior MVR	2 (1%)	0 (0%)	2 (2%)	0.155
Horizontal aorta	82 (41.0%)	47 (47%)	35 (35%)	0.083
Prior AVR	1 (0.5%)	1 (1%)	0 (0%)	0.316
Dialysis	3 (1.5%)	2 (2%)	1 (1%)	0.561
Procedure indication				
elective	190 (95%)	94 (94%)	96 (96%)	0.516
urgent	10 (5%)	6 (6%)	4 (4%)	0.516
acute	0 (0%)	0 (0%)	0 (0%)	1

**Table 2:** Baseline parameters of transthoracic echocardiography in the total study population. LVEF: left ventricular ejection function, AVA: aortic valve area, AVAi: aortic valve area indexed to the body surface area, sPAP: systolic pulmonary arterial pressure.

Echocardiographic Parameters of the Study Population	Overall (n = 200)	Cohort A (n = 100)	Cohort B (n = 100)	*p* Value
Mean LVEF	55.6 ± 13.4	55.8 ± 13.6	55.7 ± 13.1	0.961
Mean AoVmax (m/s)	4.4 ± 0.72	4.5 ± 0.7	4.35 ± 0.73	0.232
Aortic peak gradient (Hgmm)	80.6 ± 25.7	82.9 ± 25	78.5 ± 26.3	0.243
Aortic mean gradient (Hgmm)	47.8 ± 15.6	48.6 ± 14.8	47 ± 16.3	0.489
AVA (cm^2^)	0.72 ± 0.23	0.69 ± 0.23	0.74 ± 0.22	0.254
AVAi (cm^2^)	0.37 ± 0.12	0.35 ± 0.1	0.39 ± 0.12	0.064
Mitral insufficiency III or IV	39 (19.5%)	18 (18%)	21 (21%)	0.592
Tricuspid insufficiency III or IV	38 (19%)	15 (15%)	23 (23%)	0.149
sPAP ≥ 60 Hgmm	25 (12.5%)	12 (12%)	13 (13%)	0.739

**Table 3:** Detailed data of the THV size distribution of the total study population. THV: transcatheter heart valve, BAV:bicuspid aortic valve, TAV: tricuspid aortic valve. Standard size: 23, 26, and 29. Intermediate + extra size: 21.5, 24.5, 27.5, 30.5, and 32.

THV Size	Total Cohort (n = 199)			
	Overall (n = 199)	BAV (n = 36)	TAV (n = 163)	*p* Value
21.5	8	1	7	0.561
23	29	3	26	0.099
24.5	46	8	38	0.854
26	42	4	38	0.022
27.5	43	11	32	0.075
29	20	2	18	0.16
30.5	7	5	2	<0.001
32	4	2	2	0.091
Standard size	91	9	82	<0.001
Intermediate + extra size	108	27	81	<0.001
*p* value	0.227	<0.001	0.931	

**Table 4:** Procedural data of the study population. AV: aortic valve, ARI: aortic regurgitation index, LCA: left coronary artery, RCA: right coronary artery, SOV: Sinus of Valsalva, THV: transcatheter heart valve.

Variable	Overall (n = 200)
Type of anesthesia	
general	4
local	196
Access site	
percutaneous femoral	196
surgical femoral	2
subclavia	2
axillaris	0
direct aortic	0
Contrast agent	217.9 ± 94.7
Operation duration (min)	81.1 ± 26.6
Predilatation	200
Postdilatation	28
Preimpl. mean AV gradient	53.8 ± 18.4
Postimpl. mean AV gradient	5.6 ± 5.3
ARI	28.2 ± 9.4
LCA height	14.1 ± 3.03
RCA height	17.4 ± 3.05
SOV diameter—left	33.4 ± 4.72
SOV diameter—right	32.1 ± 3.72
SOV diameter—non	33.9 ± 3.97
THV implantation depth	
Left coronary side (mm)	5.5 ± 2.1
Non coronary side (mm)	6.2 ± 2.0
Right coronary side (mm)	6.0 ± 1.9
Average depth (mm)	5.9 ± 1.9
Oversize	7.0 ± 4.3
New Permanent PM impl.	54 (29.5%)

**Table 5:** Detailed data of postprocedural outcomes of the study population based on VARC-2 definition. TIA: transient ischaemic attack, AR: aortic regurgitation.

Postprocedural Outcomes < 72 h After the Index Procedure	
Outcome	Overall (n = 200)
	No. (%) of events
In-hospital mortality	2 (1%)
Device success	198 (99%)
Myocardial infarction	0 (0%)
Coronary obstruction	0 (0%)
Stroke or TIA	4 (2%)
Acute kidney injure, stage 2 or 3	7 (3.5%)
Major vascular complications	12 (6%)
Minor vascular complications	7 (3.5%)
Cardiac tamponade	0 (0%)
Annulus rupture	0 (0%)
Valve malpositioning	0 (0%)
Need for a second valve	0 (0%)
Posptocedural AR grade III or IV	0 (0%)

**Table 6:** Detailed data of comparison between patients with and without permanent pacemaker implantation in the study population. Ca score: Agatston Calcium score of the aortic valve based on CT examination, Ca in LVOT: existence of calcium nodulus in the left ventricle outflow tract based on CT examination, THV: transcatheter heart valve. *—level of statistically significance.

	Total Study Population		
	Non PM (n = 129)	PM (n = 54)	*p*-Value
Age	74.9 ± 6.9	75.3 ± 7.5	0.735
Euroscore	15.5 ± 15.9	11.6 ± 9.1	0.035 *
Euroscore II	5.4 ± 5.8	4.5 ± 3.8	0.32
STS score	5.8 ± 3.7	5.3 ± 4.2	0.465
Ca score	3288 ± 1707	3392 ± 1829	0.717
Ca in LVOT	43	25	0.049 *
Bicuspid	24 (16.4%)	11 (20.4%)	0.783
Oversizing	7.1 ± 4.7	7.0 ± 3.8	0.9
THV implantation depth			
Left coronary side (mm)	5.3 ± 2.3	5.9 ± 1.8	0.085
Right coronary side (mm)	5.8 ± 2.1	6.3 ± 1.7	0.145
Non coronary side (mm)	6.1 ± 2.2	6.4 ± 1.7	0.253
Average depth (mm)	5.7 ± 2.1	6.2 ± 1.6	0.132
THV size			
21.5	5	2	0.948
23	22	7	0.477
24.5	32	13	0.895
26	27	11	0.913
27.5	28	10	0.611
29	9	7	0.197
30.5	2	3	0.132
32	3	1	0.836
Standard size	58	25	0.903
Intermediate/extra size	70	29	0.942
*p*-value	0.134	0.441	

**Table 7:** Detailed data of postprocedural outcomes at 30-day and 1-year follow-up of the study population based on VARC-2 definition. BAV: balloon aortic valvuloplasty, TAVI: transcatheter aortic valve implantation, SAVR: surgical aortic valve replacement.

VARC-2 Outcomes at 30-Day and 1-Year Follow-Up		
Outcome	30-Day Cumulative Clinical Outcomes (n = 200)	One-Year Cumulative Clinical Outcomes (n = 200)
All-cause mortality	4 (2%)	17 (8.5%)
Cardiac mortality	1 (0.5%)	3 (1.5%)
All stroke	4 (2%)	10 (5%)
Life-threatening bleeding	6 (3%)	6 (3%)
Acute kidney injury, stage 2 or 3	4 ((2%)	4 (2%)
Coronary artery obstruction	0 (0%)	0 (0%)
Major vascular complication	12 (6%)	12 (6%)
New pacemaker implantation	56 (30.6%)	58 (31.7%)
Valve-related dysfunction requiring repeat procedure (BAV, TAVI, or SAVR)	0 (0%)	2 (1%)
Requiring hospitalizations for worsening heart failure	1 (0.5%)	2 (1%)
NYHA class III or IV	0 (0%)	2 (1%)
Valve thrombosis	0 (0%)	0 (0%)
Endocarditis	0 (0%)	4 (2%)

**Table 8:** Detailed data of the echocardiographic parameters in the study population. NA: not added value.

Echocardiography Parameters	Overall (n = 200)
Peak aortic gradient (mmHg)	
baseline	80.6 ± 25.7
discharge	20.2 ± 7.4
30-day follow-up	20.7 ± 7.6
1-year follow-up	22.5 ± 8.6
Mean aortic gradient (mmHg)	
baseline	47.8 ± 15.6
discharge	10.4 ± 4.3
30-day follow-up	10 ± 4.2
1-year follow-up	11.2 ± 4.5
Global ejection fraction (%)	
baseline	55.8 ± 13.4
discharge	56 ± 10.3
30-day follow-up	59.5 ± 10.4
1-year follow-up	60.7 ± 10.5
Aortic regurgitation grade 2 or above	
baseline	73 (36.5%)
discharge	11 (5.5%)
30-day follow-up	5 (2.5%)
1-year follow-up	5 (2.7)
Paravalvular leak grading moderate or above	
baseline	NA
discharge	1 (0.5%)
30-day follow-up	1 (0.5%)
1-year follow-up	1 (0.5%)
Mitral regurgitation grade 3 or 4	
baseline	39 (19.5%)
discharge	15 (7.6%)
30-day follow-up	14 (7.1%)
1-year follow-up	15 (8.2%)

## Data Availability

The data presented in this study are available on request from the corresponding author. The data are not publicly available due to Hungarian legal regulations.

## Supplementary Materials

The following supporting information can be downloaded at: https://www.mdpi.com/article/10.3390/jcm14072323/s1, Table S1: Vascular complication of the Cohort A and Cohort B based on the VARC-2 definition. AFC: common femoral artery; Table S2: Baseline characteristics of the total study population, in the two cohorts and the comparison of the Cohort A and Cohort B. BAV: balloon aorto-valvuloplasty. MVR: mitral valve replacement. AVR: aortic valve replacement; Table S3: Baseline parameters of transthoracic echocardiography in the total study population and in the subgroups of Cohort A (first 100 patient) and Cohort B (second 100 patient). LVEF: left ventricular ejection function, AVA: aortic valve area, AVAi: aortic valve area indexed to the body surface area. sPAP: systolic pulmonary arterial pressure; Table S4: Detailed data of the THV size distribution of the total study population and in the subgroups. BAV:bicuspid aortic valve. TAV: tricuspid aortic valve. Standard size: 23, 26, 29. Intermediate + extra size: 21.5, 24.5, 27.5, 30.5, 32; Table S5: Procedural data of the whole study population and in the subgroups of Cohort A and Cohort B. ARI: aortic regurgitation index, LCA: left coronary artery, RCA: right coronary artery, SOV: Sinus of Valsalva; Table S6: Detailed data of postprocedural outcomes of the whole study population and in the Cohort A and Cohort B, based on VARC-2 definition; Table S7: Detailed data of comparison between patients with and without permanent pacemaker implantation in the total study population and in the subgroups. Ca score: Agatston Calcium score of the aortic valve based on CT examination. Ca in LVOT: existence of calcium nodulus in the left ventricle outflow tract based on CT examination; Table S8: Detailed data of postprocedural outcomes at 30-day and 1-year follow-up of the whole study population and in the Cohort A and Cohort B, based on VARC-2 definition; Table S9: Detailed data of the echocardiographic parameters in the total study population and the comparison between Cohort A and Cohort B * Statistically significant; Figure S1: Patient distribution according to NYHA functional class regarding the Cohort A and Cohort B during the follow-up period; Figure S2: Peak and mean aortic gradients at the different time period regarding the Cohort A and Cohort B.

## Abbreviations

ACT	activated clotting time
ARI	aortic regurgitation index
AS	aortic stenosis
AV	aortic valve
AVA	aortic valve area
AVAi	aortic valve area indexed to the body surface area
AVR	aortic valve replacement
BAV	bicuspid aortic valve
BEV	balloon-expandable valve
CABG	coronary artery bypass grafting
DOAC	direct oral anticoagulant
GFR	glomerular filtration rate
IVL	intravascular lithotripsy therapy
LCA	left coronary artery
LFLG-AS	low-flow low-gradient aortic stenosis
LVOT	left ventricular outflow tract
MI	myocardial infarction
MR	mitral regurgitation
MSCT	multi-slice computed tomography
MVR	mitral valve replacement
NYHA	New York Heart Association
PCI	percutaneous coronary intervention
PLFLG-AS	paradox low-flow low-gradient aortic stenosis
PM	permanent pacemaker
PPI	permanent pacemaker implantation
PPM	patient–prothesis mismatch
PVL	paravalvular leakage
RCA	right coronary artery
SAVR	surgical aortic valve replacement
SEV	self-expandable valve
SOV	Sinus of Valsalva
sPAP	systolic pulmonary arterial pressure
STS	Society of Thoracic Surgeons
TAV	tricuspid aortic valve
TAVR	transcatheter aortic valve replacement
THV	transcatheter heart valve
TIA	transient ischemic attack
VARC-2	Valve Academic Research Consortium-2
