# Supplementary material for: Single-Centre Experience with the Balloon-Expandable Myval Transcatheter Aortic Valve System with the First 200 Patients: 30-Day and 1-Year Follow-Up
Source: J Clin Med. 2025 Mar 28;14(7):2323. doi: 10.3390/jcm14072323 (PMC11989386; doi:10.3390/jcm14072323)
Supplement: Supplementary file 1 [file jcm-14-02323-s001.zip › jcm-3442427-supplementary.pdf]

Supplementary Materials

Table S1. Vascular complication of the Cohort A and Cohort B based on the VARC-2 definition. AFC: common femoral artery.

Vascular complications of study population		
Form of vascular complication	*Type of intervention to treat*	*Amount of transfusion (U)*
	Cohort A	
major	surgical intervention of AFC	4
minor	balloon angioplasty of AFC	0
major	stent implantation of axillary artery	4
major	balloon angioplasty of AFC	2
major	device extraction via vascular surgery	6
minor	balloon angioplasty of AFC	0
minor	balloon angioplasty of AFC	0
major	balloon angioplasty of AFC	4
major	surgical intervention of AFC	4
minor	balloon angioplasty of AFC	0
minor	balloon angioplasty of subclavian artery	0
	Cohort B	
major	balloon angioplasty of AFC	2
minor	embolectomy from the brachial artery	0
major	surgical intervention of AFC	2
major	balloon angioplasty of AFC	2
minor	balloon angioplasty of AFC	0
major	balloon angioplasty of iliac artery	9
major	balloon angioplasty of AFC and stent implantation	4
major	balloon angioplasty of AFC	2

Table S2. Baseline characteristics of the total study population, in the two cohorts and the comparison of the Cohort A and Cohort B. BAV: balloon aorto-valvuloplasty. MVR: mitral valve replacement. AVR: aortic valve replacement.

Baseline characteristics of study population				
	**Overall (n=200)**	**Cohort A (n=1-100)**	**Cohort B (n=101-200)**	p value
Age (yrs)	75.3 ± 6.9	74.7 ± 7.2	75.9 ± 6.5	0.192
Male/Female	122/78	63/37	59/41	0.562
Body mass index (kg/m^2)	29 ± 5.2	29.4 ± 4.8	28.6 ± 5.6	0.242
Body surface area (m^2)	1.93 ± 0.2	1.94 ± 0.2	1.91 ± 0.2	0.223
Hypertension	195 (97.5%)	95 (95%)	100 (100%)	0.007
Diabetes mellitus	79 (39.5%)	40 (40%)	39 (39%)	0.885
Hyperlipidemia	182 (91%)	84 (84%)	98 (98%)	0.001
NYHA class I	2 (1%)	2 (2%)	0 (0%)	0.316
NYHA class II	39 (19.5%)	34 (34%)	5 (5%)	<0.001
NYHA class III	147 (73.5%)	60 (60%)	87 (87%)	<0.001
NYHA class IV	12 (6%)	4 (4%)	8 (8%)	0.39
Ischaemic Heart Disease	85 (42.5%)	47 (47%)	38 (38%)	0.198
Prior MI	50 (25%)	24 (24%)	26 (26%)	0.744
Prior PCI	70 (35%)	39 (39%)	31 (31%)	0.298
Prior CABG	36 (18%)	22 (22%)	14 (14%)	0.101
Peripheral artery disease	24 (12%)	10 (10%)	14 (14%)	0.384
Cerebrovascular disease	29 (14.5%)	8 (8%)	21 (21%)	0.009
Pulmonary disease	28 (14%)	15 (15%)	13 (13%)	0.836
Previous aortic balloon valvuloplasty	9 (4.5%)	5 (5%)	4 (4%)	0.733
Permanent PM	17 (8.5%)	9 (9%)	8 (8%)	0.8

Atrial fibrillation	41 (20.5%)	18 (18%)	23 (23%)	0.381
Logistic EuroSCORE (%)	15.2 ± 15	15.7 ±15.5	15.1 ± 14.5	0.921
Euroscore II	5.4 ± 5.4	4.8 ± 4.9	6.0 ± 5.9	0.115
STS score (%)	5.8 ± 3.8	5.6 ± 3.9	5.9 ± 3.8	0.582
Aortic valve Calcium score	3308 ± 1726	3395 ± 1832	3211 ± 1628	0.437
Serum creatinine (umol/l)	102.3 ± 48.2	102.7 ± 58.8	101.8 ± 35.0	0.857
Estimated GFR (ml/min)	67.6 ± 25.9	69.6 ± 26.6	65 ± 25.5	0.224
Estimated GFR < 60 ml/min	89 (44.5%)	40 (40%)	49 (49%)	0.2
Bicuspid aortic valve	36 (18%)	17 (17%)	19 (19%)	0.713
Small annulus (≤430 mm²)	52 (26%)	24 (24%)	28 (28%)	0.519
Prior MVR	2 (1%)	0 (0%)	2 (2%)	0.155
Prior AVR	1 (0.5%)	1 (1%)	0 (0%)	0.316
Dialysis	3 (1.5%)	2 (2%)	1 (1%)	0.561
Procedure indication				
elective	190 (95%)	94 (94%)	96 (96%)	0.516
urgent	10 (5%)	6 (6%)	4 (4%)	0.516
acute	0 (0%)	0 (0%)	0 (0%)	1

Table S3. Baseline parameters of transthoracic echocardiography in the total study population and in the subgroups of Cohort A (first 100 patient) and Cohort B (second 100 patient). LVEF: left ventricular ejection function, AVA: aortic valve area, AVAi: aortic valve area indexed to the body surface area. sPAP: systolic pulmonary arterial pressure.

Echocardiographic parameters of the study population	*Overall (n=200)*	*Cohort A (n=100)*	*Cohort B (n=100)*	**p value**
Mean LVEF	55.6 ± 13.4	55.8 ±13.6	55.7 ± 13.1	0.961
Mean AoVmax (m/s)	4.4 ± 0.72	4.5 ± 0.7	4.35 ± 0.73	0.232
Aortic peak gradient (Hgmm)	80.6 ± 25.7	82.9 ± 25	78.5 ± 26.3	0.243
Aortic mean gradient (Hgmm)	47.8 ± 15.6	48.6 ± 14.8	47 ± 16.3	0.489
AVA (cm2)	0.72 ± 0.23	0.69 ± 0.23	0.74 ± 0.22	0.254
AVAi (cm2)	0.37 ± 0.12	0.35 ± 0.1	0.39 ± 0.12	0.064
Mitral insufficiency III or IV	39 (19.5%)	18 (18%)	21 (21%)	0.592
Tricuspid insufficiency III or IV	38 (19%)	15 (15%)	23 (23%)	0.149
sPAP ≥ 60 Hgmm	25 (12.5%)	12 (12%)	13 (13%)	0.739

Table S4. Detailed data of the THV size distribution of the total study population and in the subgroups. BAV:bicuspid aortic valve. TAV: tricuspid aortic valve. Standard size: 23,26,29. Intermediate+extra size: 21.5, 24.5, 27.5, 30.5, 32.

**THV size**	*Total Cohort (n=199)*				*Cohort A (n=99)*				*Cohort B (n=100)*			
	**Overall (n=199)**	**BAV (n=36)**	**TAV (n=163)**	**p value**	**Overall (n=99)**	**BAV (n=17)**	**TAV (n=82)**	**p value**	**Overall (n=100)**	**BAV (n=19)**	**TAV (n=81)**	**p value**
21.5	8	1	7	0.561	7	1	6	0.683	1	0	1	0.265
23	29	3	26	0.099	11	2	9	0.86	18	1	17	<0.001
24.5	46	8	38	0.854	28	7	21	0.02	18	1	17	<0.001
26	42	4	38	0.022	19	1	18	0.001	23	3	20	0.117
27.5	43	11	32	0.075	16	2	14	0.285	27	9	18	<0.001
29	20	2	18	0.16	14	1	13	0.023	6	1	5	0.782
30.5	7	5	2	<0.001	2	1	1	0.075	5	4	1	<0.001
32	4	2	2	0.091	2	2	0	<0.001	2	0	2	0.114
Standard size	91	9	82	<0.001	44	4	40	<0.001	47	5	42	<0.001
Intermediate+extra size	108	27	81	<0.001	55	13	42	<0.001	53	14	39	<0.001
p value	0.227	<0.001	0.931		0.116	<0.001	0.73		0.396	<0.001	0.6	

Table S5. Procedural data of the whole study population and in the subgroups of Cohort A and Cohort B. ARI: aortic regurgitation index, LCA: left coronary artery, RCA: right coronary artery, SOV: Sinus of Valsalva.

Variable	*Overall (n=200*	*Cohort A (n=100)*	*Cohort B (n=100)*	**p value**
**Type of anesthesia**				
general	4	3	1	0.312
local	196	97	99	0.312
**Access site**				
percutaneous femoral	196	97	99	0.31
surgical femoral	2	2	0	0.16
subclavia	2	1	1	1
axillaris	0	0	0	1
direct aortic	0	0	0	1
Contrast agent	217.9 ± 94.7	225.8 ± 98.6	210.9 ± 91	0.313
Operation duration (min)	81.1 ± 26.6	80.1 ± 31.9	82.3 ± 20.4	0.517
Predilatation	200	100	100	NA
Postdilatation	28	25	3	<0.001
Preimpl. mean AV gradient	53.8 ± 18.4	55.6 ± 18.3	52.1 ± 18.3	0.187
Postimpl. mean AV gradient	5.6 ± 5.3	5.3 ± 5.7	5.9 ± 4.7	0.415
ARI	28.2 ± 9.4	27.9 ± 9.7	28.6 ± 9.1	0.619
LCA height	14.1 ± 3.03	14.2 ± 3.36	13.9 ± 2.68	0.56
RCA height	17.4 ± 3.05	17.9 ± 3.03	17.0 ± 3.02	0.032
SOV diameter - left	33.4 ± 4.72	33.4 ± 4.8	33.5 ± 4.67	0.837
SOV diameter - right	32.1 ± 3.72	31.7 ± 3.86	32.4 ± 3.56	0.227
SOV diameter - non	33.9 ± 3.97	34.0 ± 4.21	33.9 ± 3.72	0.864
THV implantation depth				
Left coronary side (mm)	5.5 ± 2.1	5.6 ± 2.3	5.5 ± 1.98	0.778
Non coronary side (mm)	6.2 ± 2.0	6.2 ± 2.3	6.2 ± 1.7	0.932
Right coronary side (mm)	6.0 ± 1.9	6.0 ± 2.2	6.0 ± 1.7	0.986
Average depth (mm)	5.9 ± 1.9	5.9 ± 2.1	5.9 ± 1.7	0.947
Oversize	7.0 ± 4.3	7.4 ± 4.0	6.6 ± 4.6	0.181
New Permanent PM impl.	54 (29.5%)	28 (30.7%)	26 (28.2%)	0.75

Table S6. Detailed data of postprocedural outcomes of the whole study population and in the Cohort A and Cohort B, based on VARC-2 definition.

Postprocedural outcomes < 72 h after the index procedure				
**Outcome**	**Overall (n=200)**	**Cohort A** (n=100)	**Cohort B** (n=100)	**p value**
		No. (%) of events		
In-hospital mortality	2 (1%)	1 (1%)	1 (1%)	1
Device success	198 (99%)	99 (99%)	99 (99%)	1
Myocardial infarction	0 (0%)	0 (0%)	0 (0%)	1
Coronary obstruction	0 (0%)	0 (0%)	0 (0%)	1
Stroke or TIA	4 (2%)	1 (1%)	3 (3%)	0.312
Acute kidney injure, stage 2 or 3	7 (3.5%)	3 (3%)	4 (4%)	0.7
Major vascular complications	12 (6%)	6 (6%)	6 (6%)	0.516
Minor vascular complications	7 (3.5%)	5 (5%)	2 (2%)	0.733
Cardiac tamponade	0 (0%)	0 (0%)	0 (0%)	1
Annulus rupture	0 (0%)	0 (0%)	0 (0%)	1
Valve malpositioning	1 (0.5%)	0 (0%)	1 (1%)	0.316
Need for a second valve	0 (0%)	0 (0%)	0 (0%)	1
Posptocedural AR grade III or IV	0 (0%)	0 (0%)	0 (0%)	1
New Permanent PM impl.	54 (29.5%)	28 (30.7%)	26 (28.2%)	0.75

Table S7. Detailed data of comparison between patients with and without permanent pacemaker implantation in the total study population and in the subgroups. Ca score: Agatston Calcium score of the aortic valve based on CT examination. Ca in LVOT: existence of calcium nodulus in the left ventricle outflow tract based on CT examination.

	**Total study population**			**Cohort A**			**Cohort B**			**Overall PM cohort**		
	**Non PM (n=129)**	**PM (n=54)**	**p-value**	**Non PM (n=62)**	**PM (n=28)**	**p-value**	**Non PM (n=66)**	**PM (n=26)**	**p-value**	**PM Cohort A (n=28)**	**PM Cohort B (n=26)**	**p-value**
**Age**	74.9 ± 6.9	75.3 ± 7.5	0.735	74.4 ± 7.0	75.8 ± 7.8	0.382	75.9 ± 6.4	74.8 ± 7.0	0.464	75.8 ± 7.8	74.8 ± 7.0	0.622
**Euroscore**	15.5 ± 15.9	11.6 ± 9.1	0.035*	17.0 ± 17.6	11.6 ± 7.9	0.035*	15.3 ± 15.7	11.3 ± 9.8	0.148	11.6 ± 7.9	11.3 ± 9.8	0.843
**Euroscore II**	5.4 ± 5.8	4.5 ± 3.8	0.32	5.2 ± 5.4	4.0 ± 3.2	0.286	6.0 ± 6.5	5.1 ± 4.5	0.525	4.0 ± 3.2	5.1 ± 4.5	0.244
**STS score**	5.8 ± 3.7	5.3 ± 4.2	0.465	5.7 ± 3.4	5.4 ± 5.1	0.681	5.9 ± 4.0	5.3 ± 2.7	0.492	5.4 ± 5.1	5.3 ± 2.7	0.988
**Ca score**	3288 ± 1707	3392 ± 1829	0.717	3332 ± 1702	3545 ± 2135	0.601	3326 ± 1722	3084 ± 1469	0.542	3545 ± 2135	3084 ± 1469	0.264
**Ca in LVOT**	43	25	*0.049**	24	13 (46.4%)	*0.455*	19	12	0.056	13 (46.4%)	12	0.984
**Bicuspid**	24 (16.4%)	11 (20.4%)	*0.783*	13 (20.9%)	4 (14.3%)	*0.462*	11	7	*0.289*	4 (14.3%)	7	0.252
**Oversizing**	7.1 ± 4.7	7.0 ± 3.8	0.9	7.8 ± 4.0	7.2 ± 3.9	0.516	6.5 ± 5.1	7.4 ± 3.6	0.447	7.2 ± 3.9	7.4 ± 3.6	0.484
**THV implantation depth**												
Left coronary side (mm)	5.3 ± 2.3	5.9 ± 1.8	0.085	5.34 ± 2.35	6.01 ± 1.99	0.197	5.4 ± 2.2	5.7 ± 1.5	0.503	6.01 ± 1.99	5.7 ± 1.5	0.461
Right coronary side (mm)	5.8 ± 2.1	6.3 ± 1.7	0.145	5.88 ± 2.25	6.2 ± 1.97	0.514	5.9 ± 1.9	6.3 ± 1.3	0.289	6.2 ± 1.97	6.3 ± 1.3	0.946
Non coronary side (mm)	6.1 ± 2.2	6.4 ± 1.7	0.253	6.11 ± 2.43	6.26 ± 2.03	0.775	6.1 ± 1.8	6.5 ± 1.4	0.255	6.26 ± 2.03	6.5 ± 1.4	0.687
Average depth (mm)	5.7 ± 2.1	6.2 ± 1.6	0.132	5.78 ± 2.25	6.16 ± 1.9	0.441	5.8 ± 1.9	6.2 ± 1.3	0.327	6.16 ± 1.9	6.2 ± 1.3	0.917
						**THV size**						
21.5	5	2	0.948	5	1	0.429	0	1	0.109	1	1	0.957
23	22	7	0.477	7	4	0.688	15	3	0.223	4	3	0.764
24.5	32	13	0.895	19	8	0.842	13	5	0.960	8	5	0.422
26	27	11	0.913	11	6	0.679	16	5	0.606	6	5	0.841
27.5	28	10	0.611	13	2	0.103	15	8	0.422	2	8	0.026
29	9	7	0.197	6	5	0.273	3	2	0.549	5	2	0.267
30.5	2	3	0.132	0	1	0.135	2	2	0.323	1	2	0.509
32	3	1	0.836	1	1	0.560	2	0	0.369	1	0	0.331
**Standard size**	58	25	0.903	24 (38.7%)	15 (53.5%)	0.188	34 (51.5%)	10 (38.5%)	0.259	15 (53.5%)	10 (38.5%)	0.266
**Intermediate/extra size**	70	29	*0.942*	38 (61.3%)	13 (46.5%)	*0.471*	32 (48.5%)	16 (61.5%)	*0.51*	13 (46.5%)	16 (61.5%)	0.272
	0.134	0.441		0.012	0.591		0.73	0.095		0.591	0.095	

Table S8. Detailed data of postprocedural outcomes at 30-day and 1-year follow-up of the whole study population and in the Cohort A and Cohort B, based on VARC-2 definition.

VARC-2 outcomes at 30-day and 1-year follow-up				
**Outcome**	**Overall (n=200)**	**Cohort A (n=100)**	**Cohort B (n=100)**	**p value**
**30-day cumulative clinical outcomes**				
All-cause mortality	4 (2%)	1 (1%)	3 (3%)	0.312
Cardiac mortality	1 (0.5%)	0 (0%)	1 (1%)	0.316
All stroke	4 (2%)	1 (1%)	3 (3%)	0.312

Life-threatening bleeding	6 (3%)	5 (5%)	1 (1%)	0.097
Acute kidney injury, stage 2 or 3	4 ((2%)	3 (3%)	1 (1%)	0.312
Coronary artery obstruction	0 (0%)	0 (0%)	0 (0%)	1
Major vascular complication	12 (6%)	6 (6%)	6 (6%)	1
New pacemaker implantation	56 (30.6%)	29 (31.8%)	27 (29.3%)	0.753
Valve-related dysfunction requiring repeat procedure (BAV, TAVI, or SAVR)	0 (0%)	0 (0%)	0 (0%)	1
Requiring hospitalizations for worsening heart failure	1 (0.5%)	0 (0%)	1 (1%)	0.316
NYHA class III or IV	0 (0%)	0 (0%)	0 (0%)	1
Valve thrombosis	0 (0%)	0 (0%)	0 (0%)	1
Endocarditis	0 (0%)	0 (0%)	0 (0%)	1
**One-year cumulative clinical outcomes**				
All-cause mortality	17 (8.5%)	7 (7%)	10 (10%)	0.447
Cardiac mortality	3 (1.5%)	2 (2%)	1 (1%)	0.561
All stroke	10 (5%)	5 (5%)	5 (5%)	1
Life-threatening bleeding	6 (3%)	5 (5%)	1 (1%)	0.097
Acute kidney injury, stage 2 or 3	4 (2%)	3 (3%)	1 (1%)	0.312
Coronary artery obstruction	0 (0%)	0 (0%)	0 (0%)	1
Major vascular complication	12 (6%)	6 (6%)	6 (6%)	1
New pacemaker implantation	58 (31.7%)	31 (34%)	27 (29.3%)	0.533
Valve-related dysfunction requiring repeat procedure (BAV, TAVI, or SAVR)	2 (1%)	1 (1%)	1 (1%)	1
Requiring hospitalizations for worsening heart failure	2 (1%)	1 (1%)	1 (1%)	1
NYHA class III or IV	2 (1%)	1 (1%)	1 (1%)	1
Valve thrombosis	0 (0%)	0 (0%)	0 (0%)	1
Endocarditis	4 (2%)	2 (2%)	2 (2%)	1

Table S9. Detailed data of the echocardiographic parameters in the total study population and the comparison between Cohort A and Cohort B * Statistically significant.

**Echocardiography parameters**	**Overall (n=200)**	**Cohort A (n=100)**	**Cohort B (n=100)**	**p value**
**Peak aortic gradient (mmHg)**				
baseline	80.6 ± 25.7	82.3 ± 24.3	78.5 ± 26.3	0.244
discharge	20.2 ± 7.4	19.5 ±7.6	20.7 ± 7.4	0.274
30-day follow-up	20.7 ± 7.6	20.2 ± 7.9	21.3 ± 7.2	0.276
1-year follow-up	22.5 ± 8.6	21.4 ± 7.5	23.5 ± 9.6	0.106
**Mean aortic gradient (mmHg)**				
baseline	47.8 ± 15.6	48.4 ± 14.6	47 ± 16.3	0.484
discharge	10.4 ± 4.3	10.2 ± 4.6	10.5 ± 4.0	0.63
30-day follow-up	10 ± 4.2	10 ± 4.6	10.1 ± 3.9	0.972
1-year follow-up	11.2 ± 4.5	10.7 ± 4.2	11.6 ± 4.7	0.187
**Global ejection fraction (%)**				
baseline	55.8 ± 13.4	55.6 ± 13.6	55.8 ± 13.1	0.924
discharge	56 ± 10.3	56.1 ± 9.8	56 ± 10.8	0.956
30-day follow-up	59.5 ± 10.4	58.1 ± 9.7	61 ± 11	0.058
1-year follow-up	60.7 ± 10.5	59.4 ± 11.1	61.9 ± 9.6	0.12
**Aortic regurgitation grade 2 or above**				
baseline	73 (36.5%)	29 (29.3%)	45 (46.4%)	0.019*
discharge	11 (5.5%)	5 (5%)	6 (6.1%)	0.756
30-day follow-up	5 (2.5%)	5 (5.1%)	0 (0%)	0.024*
1-year follow-up	5 (2.7)	4 (4.2%)	0 (0%)	0.043*
**Paravalvular leak grading moderate or above**				
baseline	NA	NA	NA	
discharge	1 (0.5%)	1 (1%)	0 (0%)	0.316
30-day follow-up	1 (0.5%)	1 (1%)	0 (0%)	0.316
1-year follow-up	1 (0.5%)	1 (0.5%)	0 (0%)	0.316

Mitral regurgitation grade 3 or 4				
baseline	39	18	21	0.592
discharge	15	9	6	0.321
30-day follow-up	14	6	8	0.579
1-year follow-up	15	6	9	0.321

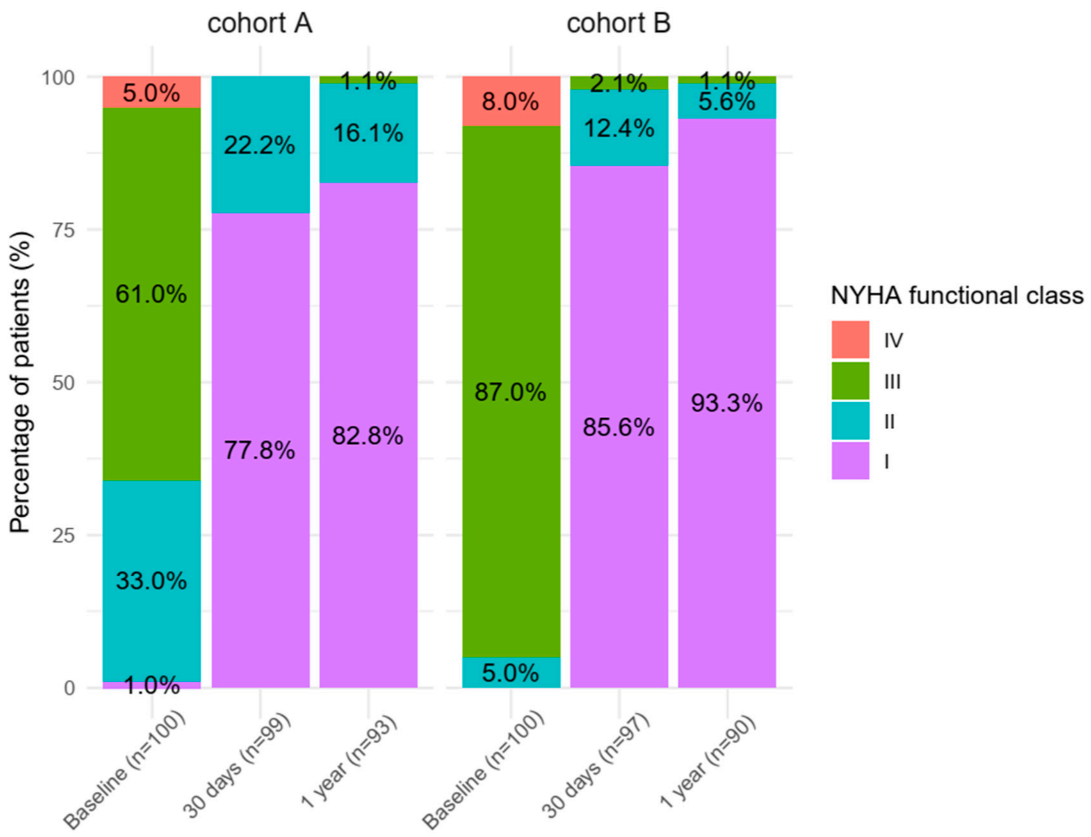

Figure S1. Patient distribution according to NYHA functional class regarding the Cohort A and Cohort B during the follow-up period.

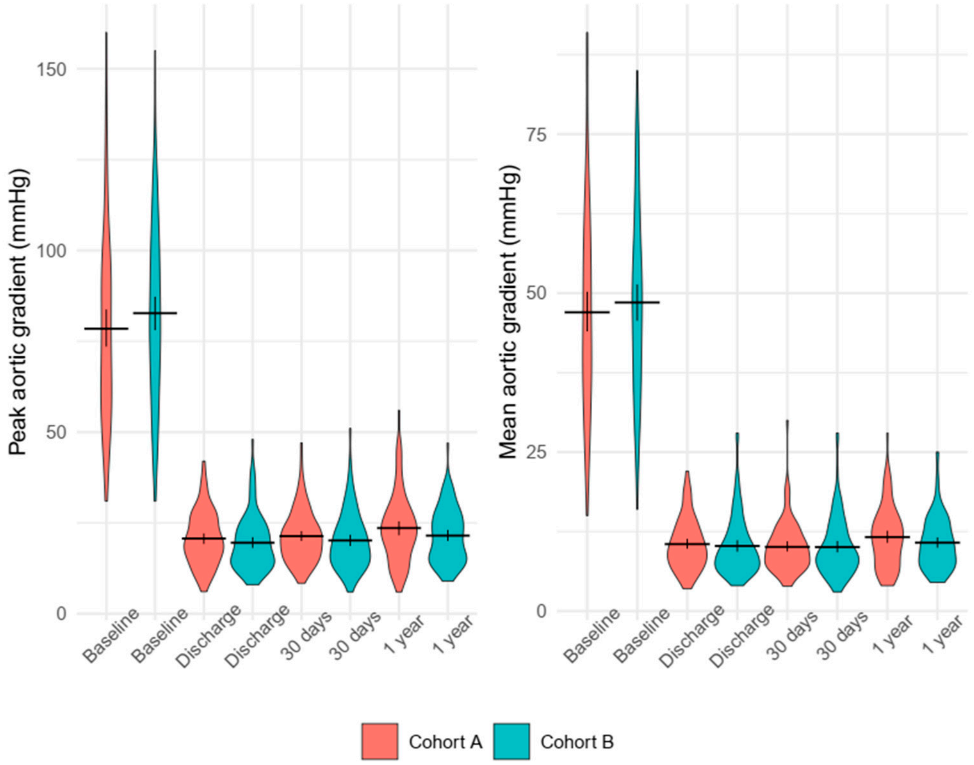

Figure S2. Peak and mean aortic gradients at the different time period regarding the Cohort A and Cohort B.
